# Identifying Immune Cell Infiltration and Effective Diagnostic Biomarkers in Rheumatoid Arthritis by Bioinformatics Analysis

**DOI:** 10.3389/fimmu.2021.726747

**Published:** 2021-08-13

**Authors:** Sheng Zhou, Hongcheng Lu, Min Xiong

**Affiliations:** ^1^Department of Orthopedics, The Affiliated Changzhou No.2 People's Hospital of Nanjing Medical University, Changzhou, China; ^2^Department of Urology, The First Affiliated Hospital of Nanjing Medical University, Nanjing, China; ^3^Department of Orthopedics, Sinopharm Dongfeng General Hospital, Hubei University of Medicine, Shiyan, China

**Keywords:** rheumatoid arthritis, synovial tissues, immune cells infiltration, diagnose biomarker, bioinformatics analysis

## Abstract

**Background:**

Rheumatoid arthritis (RA) is a chronic systemic autoimmune disorder characterized by inflammatory cell infiltration, leading to persistent synovitis and joint destruction. The pathogenesis of RA remains unclear. This study aims to explore the immune molecular mechanism of RA through bioinformatics analysis.

**Methods:**

Five microarray datasets and a high throughput sequencing dataset were downloaded. CIBERSORT algorithm was performed to evaluate immune cell infiltration in synovial tissues between RA and healthy control (HC). Wilcoxon test and Least Absolute Shrinkage and Selection Operator (LASSO) regression were conducted to identify the significantly different infiltrates of immune cells. Differentially expressed genes (DEGs) were screened by “Batch correction” and “RobustRankAggreg” methods. Functional correlation of DEGs were analyzed by Gene Ontology (GO) and Kyoto Encyclopedia of Genes and Genomes (KEGG). Candidate biomarkers were identified by cytoHubba of Cytoscape, and their diagnostic effectiveness was predicted by Receiver Operator Characteristic Curve (ROC) analysis. The association of the identified biomarkers with infiltrating immune cells was explored using Spearman’s rank correlation analysis in R software.

**Results:**

Ten significantly different types of immune cells between RA and HC were identified. A total of 202 DEGs were obtained by intersection of DEGs screened by two methods. The function of DEGs were significantly associated with immune cells. Five hub genes (CXCR4, CCL5, CD8A, CD247, and GZMA) were screened by R package “UpSet”. CCL5+CXCR4 and GZMA+CD8A were verified to have the capability to diagnose RA and early RA with the most excellent specificity and sensitivity, respectively. The correlation between immune cells and biomarkers showed that CCL5 was positively correlated with M1 macrophages, CXCR4 was positively correlated with memory activated CD4+ T cells and follicular helper T (Tfh) cells, and GZMA was positively correlated with Tfh cells.

**Conclusions:**

CCL5, CXCR4, GZMA, and CD8A can be used as diagnostic biomarker for RA. GZMA-Tfh cells, CCL5-M1 macrophages, and CXCR4- memory activated CD4+ T cells/Tfh cells may participate in the occurrence and development of RA, especially GZMA-Tfh cells for the early pathogenesis of RA.

## Introduction

Rheumatoid arthritis (RA) is a chronic systemic autoimmune disease (1). The main clinical manifestation of RA is chronic synovitis of the affected joint characterized by persistent synovitis, synovial hyperplasia, and pannus formation, which destroy the bone tissue and gradually lead to the damage of joint function (2, 3). RA is common in middle-aged women aged 40–60 years, with a prevalence rate of 0.5%–2% (4). The underlying mechanism of RA is complex, which is caused by the interaction of genetic, environmental, and immune factors, in which immune factor plays an essential role in the entire process, especially in the early stage (5, 6). Therefore, exploring the diagnostic biomarkers and revealing the immune mechanism of RA is the key to the early prevention and treatment of RA and is the focus of current research.

Immune cells in synovial membrane, including resident and infiltrating immune cells, play a vital role in the occurrence and development of RA, an autoimmune disorder (7). Studies have shown that macrophages play an important role in promoting the development of RA. These cells secrete abundant cytokines, chemokines, and degrading enzymes, which lead to joint inflammation and bone destruction. These cells can cooperate with other immune cells to aggravate the formation of arthritis (8). In the past decade, a great deal of research was conducted to understand the role of T cells, especially activated Th17 and Th1 cells, in RA (9). Autoimmune Th17 cells induce synovial stroma and innate lymphocytes to secrete the cytokine granulocyte-macrophage colony-stimulating factor (MG-CSF) to initiate and enhance autoimmune arthritis in RA (10). In addition, other immune cells, such as dendritic cells (DCs) and natural killer (NK) cells, play an important regulatory role in the pathogenesis of RA (11). However, the immune mechanisms of RA in synovium have not been investigated thoroughly. Therefore, a systematic approach is urgently needed to evaluate the contribution of immune cells and explore key genes related to immune cells.

With the development and widespread use of microarray and high-throughput sequencing technology, bioinformatics analysis can be used to identify novel genes and biomarkers for many diseases, including autoimmune disease (12–14). A previous bioinformatics study suggested that GRB10 and E2f3 could be used as diagnostic markers of osteoarthritis (15). Immune cell infiltration plays an important role in the pathogenesis of many diseases, including tumor and non-tumor diseases. Currently, cell-type identification has been utilized in many diseases; this method estimates the relative subsets of RNA transcripts (CIBERSORT) and analyzes 22 immune cell subsets in complex tissues by normalized bulk transcriptome profiles (16). Thus, this method can help us systematically explore immune cell infiltration in RA synovial tissues.

In this study, CIBERSORT was performed to analyze immune cell infiltration in RA by using five microarray datasets. Wilcoxon test and LASSO regression were conducted to identify the significantly different infiltrates of immune cells in RA and HC. After DEGs were screened, functional correlation was analyzed by GO and KEGG, and hub genes were identified by R package “UpSet.” ROC logistic regression was conducted to analyze the predictive of biomarkers. Spearman’s rank correlation in R software was also used to analyze the correlation between biomarkers and significantly different infiltrates of immune cells. This work not only systematically analyzed the infiltration of immune cells in the synovial membrane of RA but also screened novel and effective diagnostic biomarkers for RA. Results provide a new method for the diagnosis and treatment of RA.

## Materials and Methods

### GEO Dataset Collection

Gene expression profiling of RA was screened using the GEO (http://www.ncbi.nlm.nih.gov/geo) database. The inclusion criteria were as follows: (1) expression profiling by array or high-throughput sequencing of mRNA; (2) availability of the synovial tissues of patients with RA or HC from joint in the datasets; and (3) ten or more synovial specimens in the dataset. Six eligible datasets were selected, including GSE1919, GSE12021, GSE55235, GSE55457, GSE77298, and GSE89408. The details of all data are shown in **Table 1**.

**Table 1:** Basic information of selected datasets.

GEO	Platform	Tissue	Samples (number)			Experiment	Attribute	Author/Reference
		(Homo sapiens)	Total	HC	RA	type		
GSE1919	GPL91	Synovium	10	5	5	Array	Test	U. Ungethuem (17)
GSE12021	GPL96	Synovium	21	9	12	Array	Test	R. Huber (18)
GSE55235	GPL96	Synovium	20	10	10	Array	Test	D. Woetzel (19)
GSE55457	GPL96	Synovium	23	10	13	Array	Test	D. Woetzel (19)
GSE77298	GPL570	Synovium	23	7	16	Array	Test	M.G. Broeren (20)
GSE89408	GPL11154	Synovium	180	28	57/95^※^	RNA-Seq	Validation	Y. Guo (21)

^※^Means 57 early RA and 95 established RA in GSE89408 dataset.

### Data Preprocessing and Study Design

Raw and series matrix files of the five test microarray datasets, namely, GSE1919, GSE12021, GSE55235, GSE55457, and GSE77298, were downloaded. For raw data, probe expression matrix was extracted and normalized by Robust Multiarray Average (RMA) based on R package “affy.” Platform annotation file was used to convert the probe expression matrix into a gene expression matrix. For the case of multiple probes corresponding to one gene, the average value was obtained. After five gene matrixes were merged by Perl script, R package “sva” was applied to eliminate heterogeneity caused by different experimental batches and platforms. Finally, we obtained one merging normalized gene expression matrix and used R package “limma” for analysis. For series matrix files, Perl script was used to extract each probe expression matrix one by one and then convert them into gene expression matrices, respectively. R package “limma” was used to analyze each gene expression matrix. The validation RNA-Seq dataset, GSE89408, was downloaded by the form of gene expression matrix. R package “edgeR” was employed for subsequent analysis. The flow diagram of this study is shown in **Supplementary Figure S1**.

### Evaluation of Immune Cell Subtype Distribution

CIBERSORT algorithm was performed to evaluate immune cell infiltration in synovial tissues between RA and HC. This algorithm can transform the normalized gene expression matrix into the composition of infiltrating immune cells. After data were submitted to the CIBERSORT web portal (http://CIBERSORT.stanford.edu/), LM22 was used as a reference expression signature with 1000 permutations. The LM22 signature matrix defined 22 infiltrating immune cell components, including subsets of macrophages (M0 macrophages, M1 macrophages, and M2 macrophages), T cells (CD8+ T cells, naïve CD4+ T cells, memory resting CD4+ T cells, memory activated CD4+ T cells, Tfh cells, regulatory T cells, and gamma delta T cells), natural killer (NK) cells (resting NK cells and activated NK cells), mast cells (resting mast cells and activated mast cells), B cells (naïve B cells and memory B cells), dendritic cells (resting dendritic cells and activated dendritic cells), monocytes, plasma cells, neutrophils, and eosinophils. The p-values and root mean squared errors were determined for each expression file in CIBERSORT. Only data with a CIBERSORT p value < 0.05 was filtered and reserved for the following analysis. The output was directly integrated to generate an entire matrix of immune cell fractions. The results from CIBERSORT were visualized using the R packages “corplot”, “vioplot”, “ggplot2”, and “glment”.

### Principal Component Analysis (PCA)

Intra-group data repeatability in each group was verified by Pearson’s correlation test. The intra-group data repeatability of the dataset was tested by sample clustering analysis. Statistical analysis was performed by R programming language, and the results were presented by R package “ggplot2”.

### Screening of DEGs

Two methods were performed to obtain accurate DEGs from multiple microarray datasets. The first method (“Batch correction”) merged the downloaded five raw datasets into an expression matrix and then analyzed the DEGs with R package “limma” after batch correction and normalization. The second method (“RobustRankAggreg, RRA”) used the R package “limma” to analyze the DGEs of the downloaded gene expression matrices. The DEGs of each dataset were integrated with R package “RobustRankAggreg.” DEGs obtained by the two methods were intersected by Venn diagram to extract final DEGs. The threshold points for DEGs were adj.P.Val < 0.05 and |log fold change (FC)| > 1.

### Functional Enrichment Analysis

The gene names of DEGs were converted to gene ID by R package “org.Hs.eg.db”. Analyses of Gene Ontology (GO) and Kyoto Encyclopedia of Genes and Genomes (KEGG) for DEGs were performed by R package “clusterProfiler.” The significantly different GO terms and signal pathways were screened by the threshold p value <0.05 and q value <0.05. The results were visualized by R package “enrichplot” and “ggplot2”.

### Screening of Hub Genes

STRING (https://string-db.org) was performed to analyze the PPI network of DEGs with a highly reliable filtering condition (score>0.7). The interaction file (string_interactions.tsv) was downloaded. Perl was conducted to obtain the network file. The cytoHubba of Cytoscape (v 3.7.2) was conducted to score each node gene by top 10 algorithms, namely, MCC (Maximal Clique Centrality), DMNC (Density of Maximum Neighborhood Component), MNC (Maximum Neighborhood Component), Degree, EPC (Edge Percolated Component), BottleNeck, EcCentricity, Closeness, Radiality, and Betweenness. The top 60 node genes scored by each algorithm were used to screen hub genes by R package “UpSet”. The RNA-Seq dataset GSE89408 was used to validate hub genes.

### Analysis of the Predictive Value of Biomarkers

ROC analysis was performed to predict the diagnostic effectiveness of biomarkers by SSPA Statistics 23. The area under the ROC curve (AUC) value was utilized to determine the diagnostic effectiveness in discriminating RA from control samples in the GSE89408 dataset.

### Correlation Analysis Between Biomarkers and Immune Cells

The association of the identified gene biomarkers with the levels of infiltrating immune cells was explored using Spearman’s rank correlation analysis in R software. The resulting associations were visualized using the chart technique with the “ggplot2” package.

## Results

### Immune Cell Infiltration in RA and Normal Synovial Tissues

**Figure 1:**
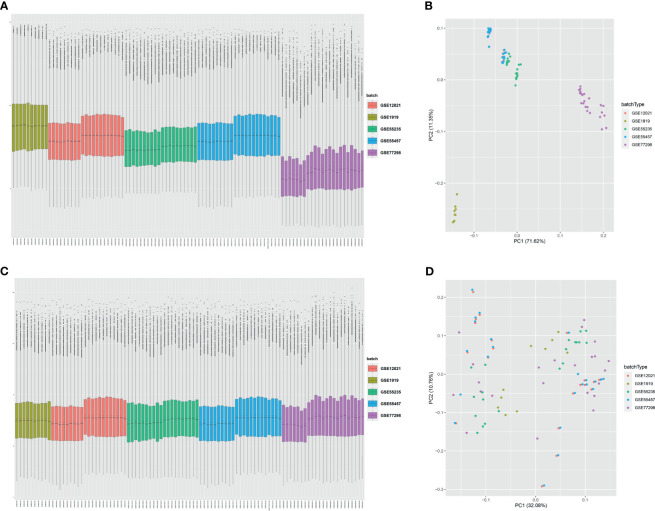
Data preprocessing. Box plot and principal component analyses were performed to remove batch correction of GSE1919, GSE12021, GSE55235, GSE55457, and GSE77298. **(A, B)** before batch correction and **(C, D)** after batch correction.

**Figure 2:**
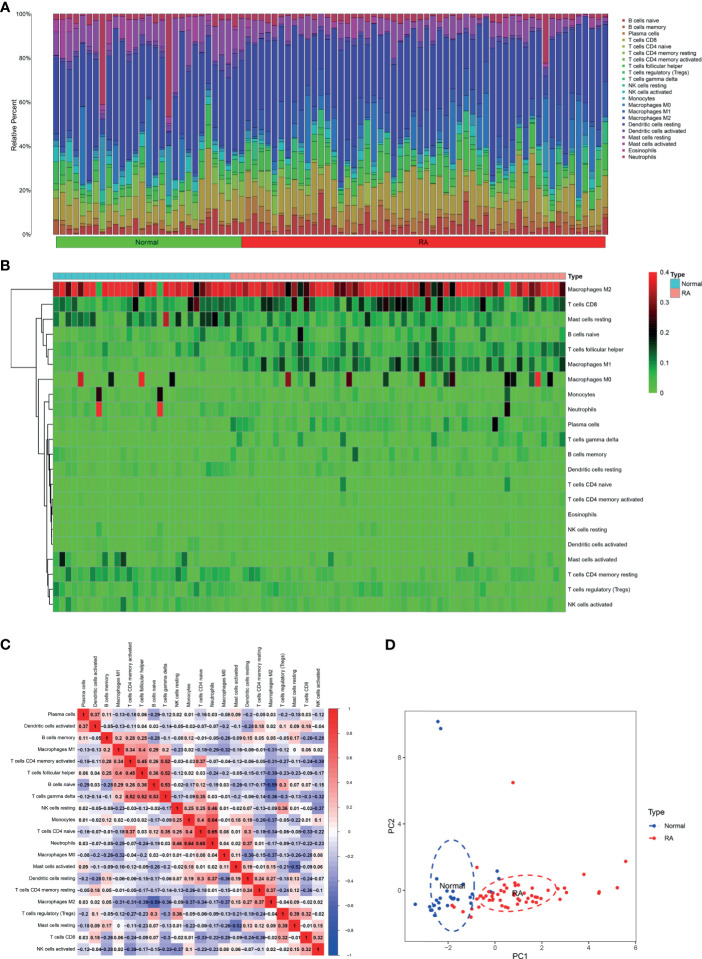
Immune cell infiltration in RA and normal synovial tissues. The composition of 22 kinds of immune cells in each sample was showed in a histogram **(A)** and a heatmap **(B)**. **(C)** The correlation of 22 types of immune cells in RA synovial tissues was evaluated. Red: positive correlation; blue: negative correlation. **(D)** PCA analysis was performed to classify infiltrating immune cells between RA and normal synovial tissues.

**Figure 3:**
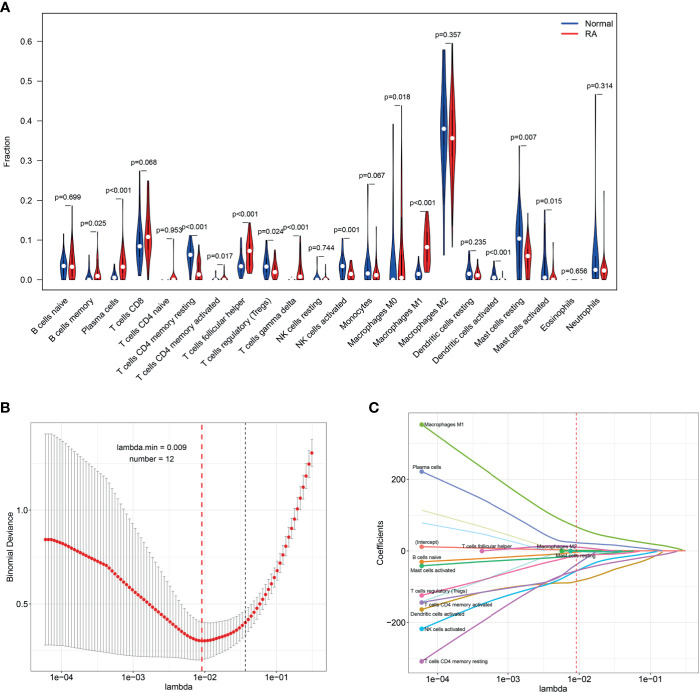
Identifying the significantly different infiltrates of immune cells in RA. **(A)** Wilcoxon test and **(B, C)** LASSO regression were conducted to analyze the different infiltrates of immune cells in RA and HC.

Five microarray raw datasets, including 56 RA and 41 normal synovial tissues, were selected for the study of immune cell infiltration. The data before (A and B) and after (C and D) batch correction was presented in **Figure 1**, which indicated that the batch effect of the merged data was removed successfully. A total of 55 RA and 29 normal synovial tissues were found to be eligible for the analysis of CIBERSORT (p < 0.05). First, the composition of 22 kinds of immune cells in each sample was presented in a histogram (**Figure 2A**) and a heatmap (**Figure 2B**). In the histogram, the color represents the percentage of different immune cells in each sample, and the sum is 1. In the heatmap, immune cells in each sample are shown with the normalized absolute abundance. The results indicated that M2 macrophages, CD8+ T cells, resting mast cells, naïve B cells, Tfh cells, M0 macrophages, and M1 macrophages were the main infiltrating immune cells. The correlation of 22 types of immune cells in RA synovial tissues was then evaluated (**Figure 2C**). For example, Tfh cells were positively correlated with memory activated CD4+ T cells and M1 macrophages. γδ T cells were positively related to memory activated CD4+ T cells and naïve B cells. Neutrophils were positively associated with resting NK cells, monocytes, and naïve CD4+ T cells. M2 macrophages were negatively correlated with naïve B cells. However, the above correlation of immune cells decreased or disappeared in HC (**Supplementary Figure S2**). Third, based on immune cell infiltration in synovial tissues, we could completely distinguished RA from normal HC by PCA analysis (**Figure 2D**). Two different algorithms, namely, Wilcoxon test and LASSO regression, were used to identify the significantly different infiltrates of immune cells in RA and HC. The results of Wilcoxon test are shown in a violin diagram in **Figure 3A**, which presented 13 types of immune cells with p < 0.05. The results of Lasso are presented in **Figures 3B, C**, which contained 12 significantly different types of immune cells. The 10 intersecting immune cells of the two methods were extracted. Compared with HC M1 macrophages, Tfh cells, the numbers of memory activated CD4+ T cells, and plasma cells were significantly higher in RA synovial tissues, while those of regulatory T cells, activated dendritic cells, activated NK cells, memory resting CD4+ T cells, resting mast cells, and activated mast cells were significantly lower in RA synovial tissues.

### Identification of DEGs

**Figure 4:**
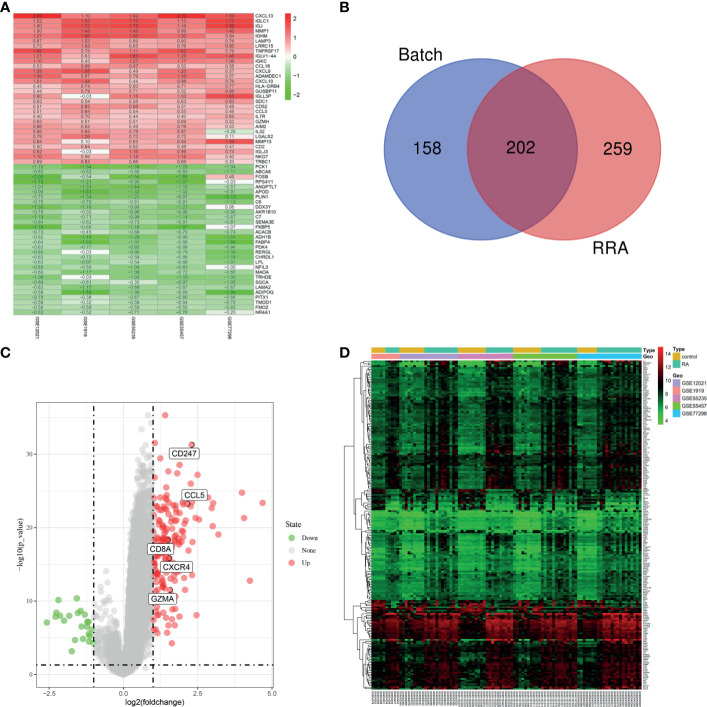
Identification of DEGs. **(A)** First 30 upregulated and downregulated DEGs of the five datasets determined by “RRA”. **(B)** Venn diagram was conducted to obtain the intersection of the DEGs screened by the two methods. The final DEGs were visualized by the volcano map **(C)** and heatmap **(D)**.

Five microarray datasets, including 57 RA and 41 normal samples, were used by the two methods to identify DEGs. A total of 360 DEGs were obtained by “Batch correction” and included 335 upregulated and 25 downregulated genes. A total of 461 DEGs were obtained by “RRA” and included 298 upregulated and 163 downregulated genes. Some of DEGs are shown in **Figure 4A**. A total of 202 DEGs, including 179 upregulated and 23 downregulated genes, were obtained through the intersection of the DEGs screened by the two methods (**Figure 4B** and **Supplementary Table S1**). The final DEGs were visualized by the volcano map (**Figure 4C**) and heatmap (**Figure 4D**).

### Functional Correlation Analysis

**Figure 5:**
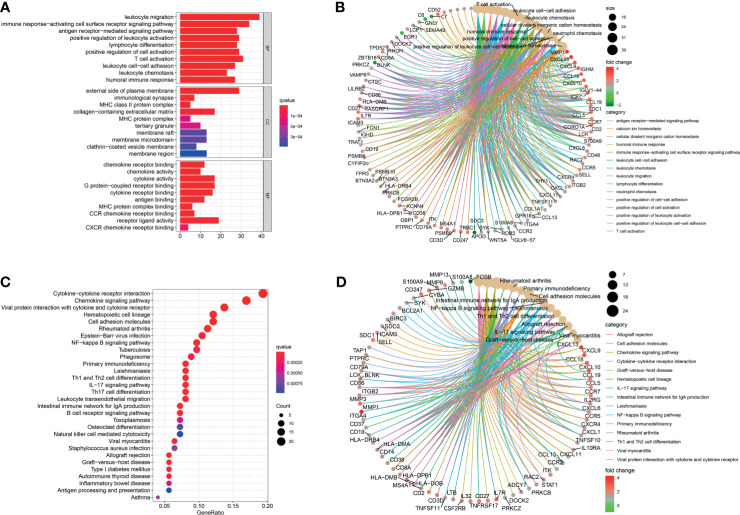
Functional correlation analysis. DEGs were analyzed by GO and KEGG. **(A, B)** The results of GO were presented by bar plot and circle charts. **(C, D)** The results of KEGG were shown by bubble and circle graphs.

After being converted into gene ID, DEGs were analyzed by GO and KEGG. The GO annotations of DEGs consisted of three parts including CC (cellular component), BP (biological process), and MF (Molecular function), which were used to analyze the functional enrichment of DEGs. The DEGs were mainly related to the biological activity of immune cells, such as lymphocyte differentiation, T cell activation, and leukocyte (**Figures 5A, B** and **Supplementary Table S2**). KEGG analysis was conducted to determine the relationship between DEGs and signaling pathway. The DEGs were mainly associated with immune cell-related signaling pathway, such as chemokine signaling pathway, rheumatoid arthritis, and primary immunodeficiency (**Figures 5C, D** and **Supplementary Table S3**). Overall, the function of DEGs were significantly associated with immune cells.

### Identification and Validation of Hub Genes

**Figure 6:**
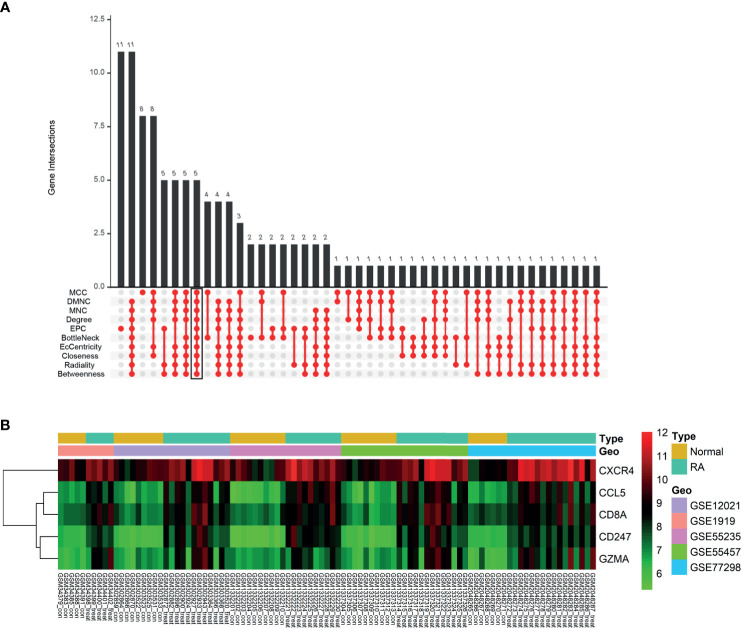
Identification of hub genes. **(A)** Ten algorithms to screen hub genes by R package “UpSet”. **(B)** Expression of five hub genes was presented by heatmap in merged microarray data.

**Figure 7:**
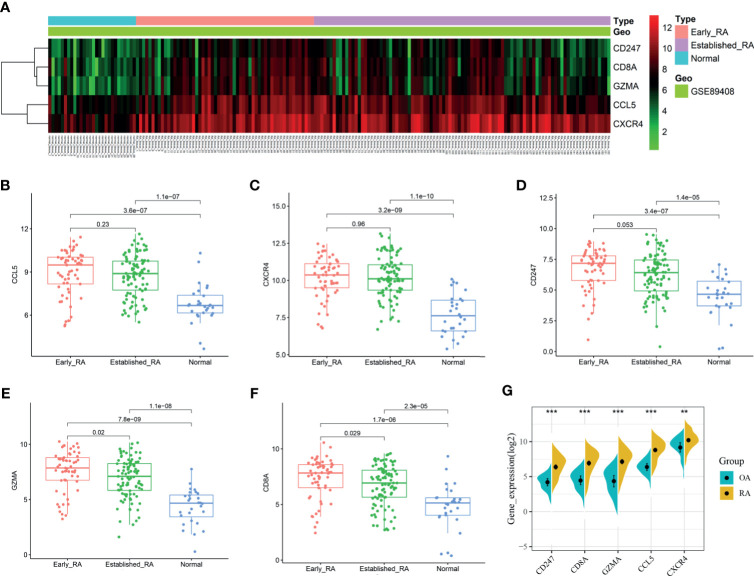
Validation of hub genes. RNA-Seq dataset GSE89408 was used to validate the expression of CXCR4, CCL5, CD8A, CD247, and GZMA, the results of which were presented as heatmap **(A)**. **(B–F)** Detailed expression of five hub genes in early RA, established RA, and HC. **(G)** Detailed expression of five hub genes in RA and HC. **p < 0.01, ***p < 0.001.

We obtained the PPI network results of DEGs by STRING (**Supplementary Figure S3**). We then used 10 algorithms to calculate the score of each node gene. Finally, we screened five hub genes (CXCR4, CCL5, CD8A, CD247, and GZMA) by R package “UpSet” and marked them with boxes in **Figure 6A**. The expression levels of the five hub genes were presented by heatmap in merged microarray data (**Figure 6B**). To make the results more reliable, we used the RNA-Seq dataset GSE89408 for validation. The expression levels of CXCR4, CCL5, CD8A, CD247, and GZMA were presented in heatmap (**Figure 7A**). As shown in **Figures 7B–F**, all the five genes had significantly higher expression in RA, both early and established RA, than in the HC (p <0.05). The expression levels of GZMA and CD8A in early RA were significantly higher than those in established RA (p <0.05). Consistent with the above results, the expression of CXCR4, CCL5, CD8A, CD247, and GZMA were also significantly increased in RA compared with osteoarthritis (OA) in dataset GSE89408 (**Figure 7G**). Therefore, GZMA and CD8A might be used as the diagnostic biomarkers for early RA, and CXCR4, CCL5, and CD247 might be used as the diagnostic biomarkers for RA.

### Diagnostic Effectiveness of Biomarkers for RA

**Figure 8:**
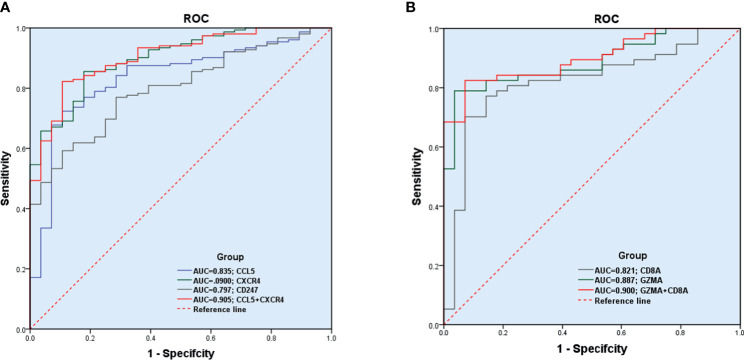
Diagnostic effectiveness of the biomarkers for RA. **(A, B)** The GSE89408 dataset was used to validate the diagnostic effectiveness of the biomarkers for RA by ROC analysis.

The GSE89408 dataset was used to validate the diagnostic effectiveness of the biomarkers for RA by ROC analysis. AUC more than 0.800 was considered as having the capability to diagnose RA with excellent specificity and sensitivity. As shown in **Figure 8A**, the AUC values of CCL5, CXCR4, and CD247 were 0.835 (95% CI 0.758–0.913), 0.900 (95% CI 0.847–0.953), and 0.797 (95% CI 0.724–0.871), respectively. Moreover, the combined AUC of CCL5 and CXCR4 reached 0.905 (95% CI 0.852–0.957). As shown in **Figure 8B**, the AUC values of GZMA and CD8A were 0.887 (95% CI 0.819–0.956) and 0.821 (95% CI 0.725–0.918), respectively. The combined AUC of GZMA and CD8A reached 0.900 (95% CI 0.837-0.963). Therefore, CCL5+CXCR4 and GZMA+CD8A had the capability to diagnose RA and early RA with excellent specificity and sensitivity, respectively.

### Correlation Between Biomarkers and Differential Immune Cells in RA

**Figure 9:**
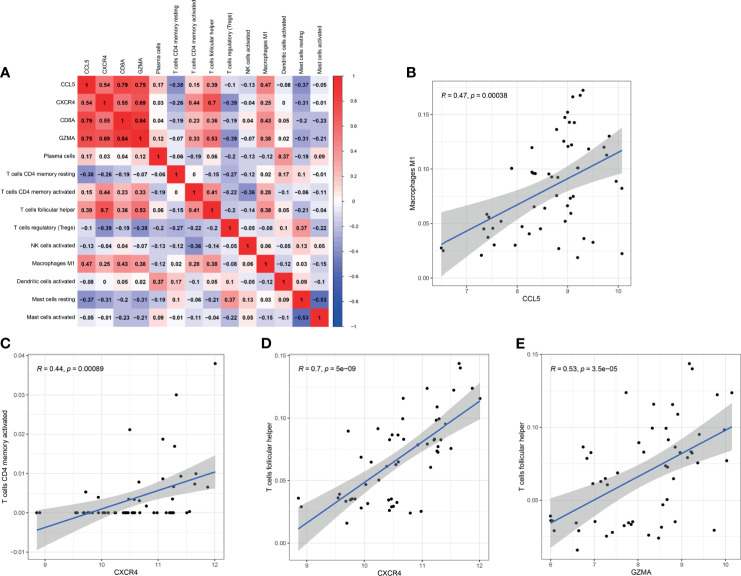
Correlation between biomarkers and differential immune cells in RA. **(A)** Correlation among four effective biomarkers and 10 significantly differential immune cells. **(B–E)** Significantly related biomarkers and immune cells by R > 0.40 and p < 0.001.

The correlation among four effective biomarkers (CCL5, CXCR4, GZMA, and CD8A) and 10 significantly differential immune cells (M1 macrophages, Tfh cells, memory activated CD4+ T cells, plasma cells, regulatory T cells, activated dendritic cells, activated NK cells, memory resting CD4+ T cells, resting mast cells, and activated mast cells) were analyzed in RA synovial tissues. The correlation results are presented in **Figure 9A**. Significantly related biomarkers and immune cells were screened by R > 0.40 and p < 0.001. The results indicated that CCL5 was positively correlated with M1 macrophages (R = 0.47, p = 0.00038), CXCR4 was positively correlated with memory activated CD4+ T cells (R = 0.44, p = 0.00089) and Tfh cells (R = 0.70, p = 5e-09), and GZMA was positively correlated with Tfh (R = 0.53, p = 5e-05) (**Figures 9B–E**).

## Discussion

RA is a systemic autoimmune disease characterized by synovitis of joints. Innate and adaptive immune responses play an indispensable role in the pathogenesis of RA. Increasing number of studies have shown that the complex interaction and activation of infiltrating immune cells are key factors in the formation of synovitis and persistent joint damage. Effective biomarkers, especially for the early stage, have not been established due to the significant heterogeneity of RA. Early diagnosis and treatment of RA could effectively prevent the disease progression of 90% patients (22). Therefore, scholars have focused on exploring the immune pathogenesis of RA and searching for novel effective biomarkers. In this study, we used comprehensive, objective, and effective bioinformatics methods to explore the role of immune cell infiltration in the synovium and identify effective diagnostic biomarkers for RA.

Compared with normal control M1 macrophages, the numbers of Tfh cells, memory activated CD4+ T cells, and plasma cells were significantly higher in RA synovial tissues, while those of regulatory T cells, activated dendritic cells, activated NK cells, memory resting CD4+ T cells, resting mast cells, and activated mast cells were significantly lower in RA synovial tissues. Macrophages can be polarized into M1 and M2 macrophages under different conditions due to their high degree of heterogeneity and plasticity. In the synovial environment of RA, macrophages mainly differentiate into type M1, which plays a pro-inflammatory role (23). The pathogenicity of M1 cells in RA is mainly realized by secreting cytokines, which in turn promote inflammation by monocyte/neutrophil recruitment, T cell polarization, and synovial fibroblast proliferation and activation (24). Tfh is a subtype of CD4+T cells, whose main functions are assisting B cells and regulating the production of antibodies (25). Tfh cell surface and secreted molecules, including CXCR5, ICOS (costimulatory molecule), and PD1 (programmed death factor 1), are involved in the development of RA (26, 27). Previous studies suggested that mast cells were aberrantly regulated in RA synovium and were mainly activated by TLRs (toll-like receptors), PAMP (pathogen-associated molecular patterns), and FcγR (Fc gamma receptor) (28, 29). Moreover, dendritic cells, NK cells, and regulatory T cells were demonstrated to play a significant role in the development of RA (30, 31). Therefore, the present results are consistent with previous reports and highlights the importance of these cells in the pathogenesis of RA by bioinformatic analysis.

To improve the accuracy of the results, the DEGs were screened by two independent methods. Further analysis indicated that the function of these DEGs were significantly associated with immune cells. Finally, five hub genes (CXCR4, CCL5, CD8A, CD247, and GZMA) were screened, which were further verified by the validation dataset (GSE89408). Compared with osteoarthritis, all the five hub genes were also significantly higher expressed in RA, which made our result more convincing. Surprisingly, the expression levels of GZMA and CD8A were significantly higher in early RA than in established RA. Therefore, GZMA and CD8A might play an important role in the pathogenesis of early RA, while CXCR4, CCL5, and CD247 might play a vital role in the overall pathogenesis of RA. ROC regression analysis further found that CCL5+CXCR4 and GZMA+CD8A had the capability to diagnose RA and early RA with excellent specificity and sensitivity, respectively.

GZMA is a member of the granzyme family and is mainly secreted by cytotoxic cells (32). In addition to its cytotoxic activity against tumor and virus-infected cells together with perforin, GZMA is involved in innate host during inflammatory and autoimmune disorders in the absence of perforin (33). However, no data have identified the actual function of GZMA. Previous studies reported the elevated expression of GZMA in plasma, synovial fluid, and synovial tissue, indicating that GZMA might be involved in the development of RA (34–36). A recent research further suggested that GZMA contributed to the joint destruction in RA in part by promoting osteoclast differentiation (37). The latest mechanism research showed that GZMA from cytotoxic lymphocytes cleaves GSDMB to trigger pyroptosis in target cells (38). We also found that GZMA was mainly highly expressed in early RA. Therefore, GZMA might be used as a diagnostic biomarker because of its essential role in the pathogenesis in early RA. CD8A encodes the CD8α chain of the dimeric CD8 protein, which is mainly involved in cell-mediated immune defense and T-cell development (39, 40). CD8a could act as diagnosis and prognosis biomarker for many diseases, including tumors and inflammatory diseases (41–44). In the present study, CD8A was significantly overexpressed in RA, especially in early RA. The ROC regression analysis further demonstrated that the combination of GZMA and CD8A had the most excellent specificity and sensitivity for diagnosis of early RA. CCL5, belonging to the C-C chemokine family, is mainly secreted by T lymphocytes, macrophages, certain types of tumor cells, and synovial fibroblasts and mainly induces the recruitment and migration of immune cells to inflammatory sites (45, 46). CXCR4, which belongs to the family of 7-trasmembrance receptors, is involved in many pathophysiological processes by binding to CXCL12 and other non-specific ligands (47). Although numerous studies indicated that CCL5 and CXCR4 played an important role in the progression of many diseases, including chronic liver disease, malignant tumor, and autoimmune disease (48–50), the specific mechanism needs to be further explored. Our study also suggests that CCL5 and CXCR4 are the hub genes of the entire process of RA development and might be used as the diagnose biomarker for this disease.

Given the important role of immune infiltrating cells and hub genes in RA, the correlation among four effective biomarkers (CCL5, CXCR4, GZMA and CD8A) and the top 10 significantly differential immune cells was further investigated in RA. CCL5 was positively correlated with M1 macrophages (R =0.47, p =0.00038), and CXCR4 was positively correlated with memory activated CD4+ T cells (R =0.44, p =0.00089) and Tfh cells (R =0.70, p =5e-09). Previous research revealed that CCL5 could directly activate M1 polarization, and CXCR4 had the ability to active memory activated CD4+ T cells and Tfh cells (51–53). Therefore, CCL5 and CXCR4 may participate in the occurrence and development of RA by regulating corresponding immune cells, which should be verified by further experiments. The most intriguing result was that GZMA was positively correlated with follicular helper T cells (R =0.53, p =5e-05). Studies have elucidated that Tfh cells and GZMA play a crucial role in infection and autoimmune diseases (54–56). M. Perreau et al. showed that HIV-infected individuals had a significantly higher frequency of Tfh cells among total CD4+ T cells compared with non-infected controls (57). Exaggerated expansion of Tfh cells resulted in self-reactive B cell proliferation, and increased long-lived plasma cell differentiation, as well as an overproduction of pathogenic autoantibodies (58). Repeated exposure to exogenous, endogenous, or symbiotic viruses and bacteria can lead to the high levels of pathogenic autoantibodies, which act as a trigger to promote the occurrence of RA (3). Previous studies have shown that Tfh and GZMA participate in the pathogenesis of RA. Our results also indicated that GZMA was significantly overexpressed in early RA. Therefore, we speculate that infectious agents may play an important role in the early pathogenesis of RA through GZMA-Tfh cells axis, which needs further experimental verification.

This research has some limitations. First, the study lacks clinically relevant information, including the activity of the disease, and the use of drugs for the disease. Second, this study was conducted only from the perspective of gene transcriptome and lacks multi-group trials. Last, only bioinformatics methods were used for data analysis, and subsequent confirmatory experiments *in vivo* and *in vitro* are needed.

In summary, our study not only offers insights into the landscape of immune cells associated with RA but also identifies effective diagnostic biomarkers for RA. GZMA-Tfh cells, CCL5-M1 macrophages, and CXCR4- memory activated CD4+ T cells/Tfh cells may participate in the occurrence and development of RA; in particular, GZMA-Tfh cells may be involved in the early pathogenesis of RA. Therefore, this study may provide a new perspective for the diagnosis and immune cellular-molecular mechanism of RA.

## Data Availability Statement

Publicly available datasets (GSE1919, GSE12021, GSE55235, GSE55457, GSE77298 and GSE89408) were analyzed in this study. All the datasets were obtained from the GEO (http://www.ncbi.nlm.nih.gov/geo) database.

## Author Contributions

SZ analyzed and wrote the manuscript. HL designed the experiments and analyzed the data. MX devised the concept and supervised the study. All authors contributed to the article and approved the submitted version.

## Conflict of Interest

The authors declare that the research was conducted in the absence of any commercial or financial relationships that could be construed as a potential conflict of interest.

## Publisher’s Note

All claims expressed in this article are solely those of the authors and do not necessarily represent those of their affiliated organizations, or those of the publisher, the editors and the reviewers. Any product that may be evaluated in this article, or claim that may be made by its manufacturer, is not guaranteed or endorsed by the publisher.

## Supplementary Material

Supplementary Figure 1 The flow diagram of this study.

Supplementary Figure 2 The correlation of 22 types of immune cells in HC synovial tissues was evaluated.

Supplementary Figure 3 PPI network results of DEGs by STRING.
