# Supplementary figures and images for: Identifying Immune Cell Infiltration and Effective Diagnostic Biomarkers in Rheumatoid Arthritis by Bioinformatics Analysis
Source: Front Immunol. 2021 Aug 13;12:726747. doi: 10.3389/fimmu.2021.726747 (PMC8411707; doi:10.3389/fimmu.2021.726747)

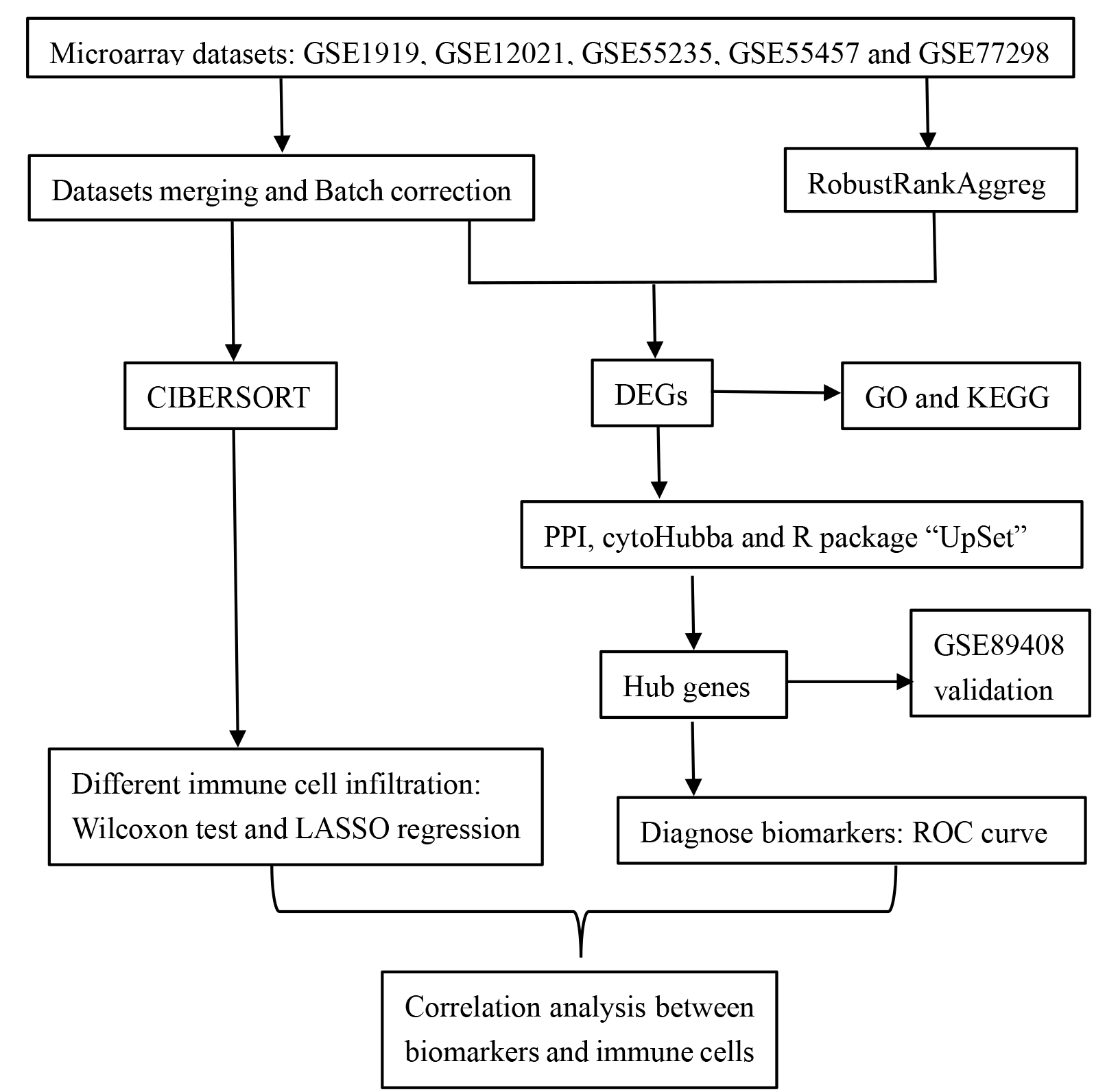

Supplement: Supplementary Figure 1 — The flow diagram of this study. [file Image_1.tif]

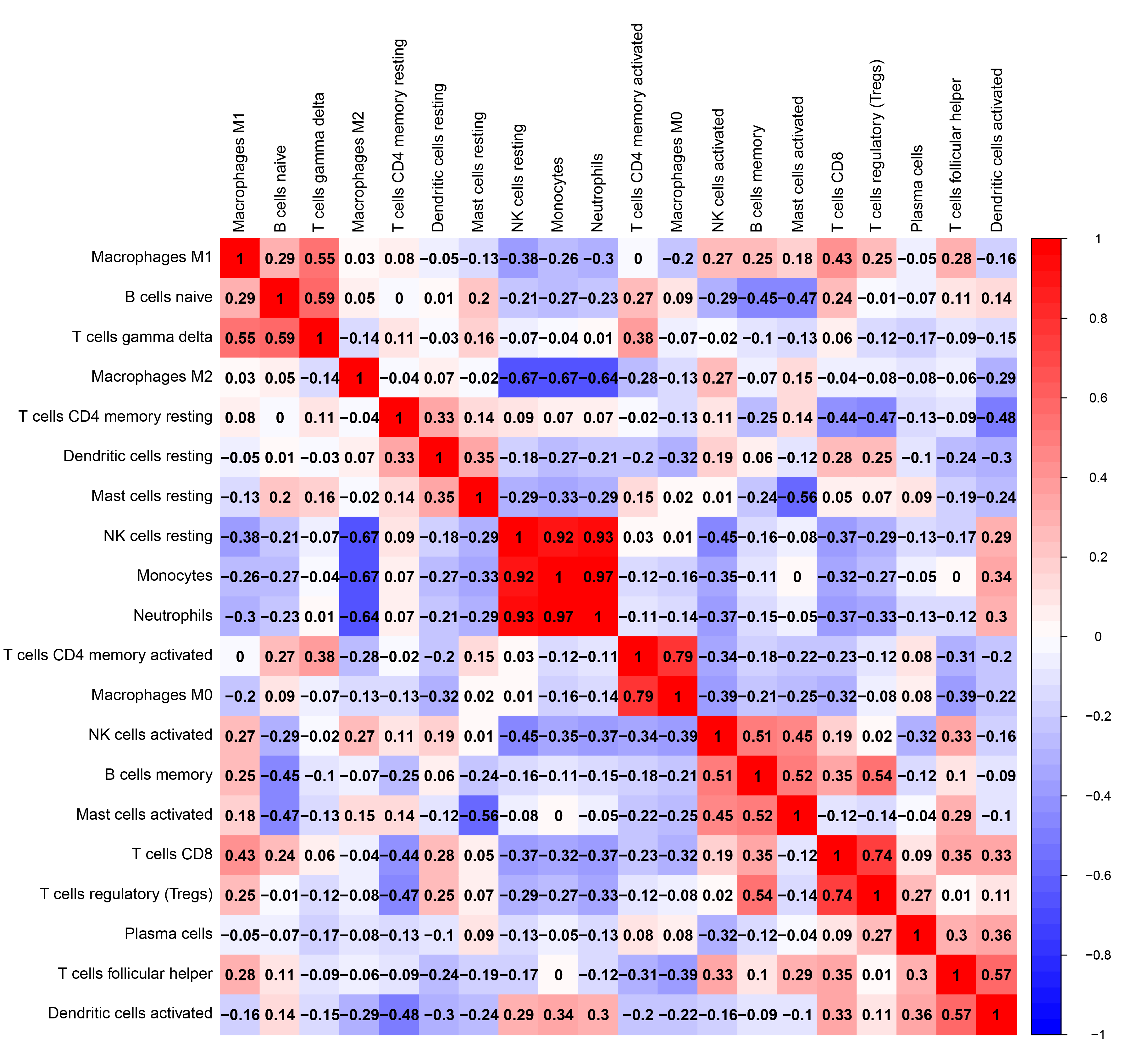

Supplement: Supplementary Figure 2 — The correlation of 22 types of immune cells in HC synovial tissues was evaluated. [file Image_2.tif]

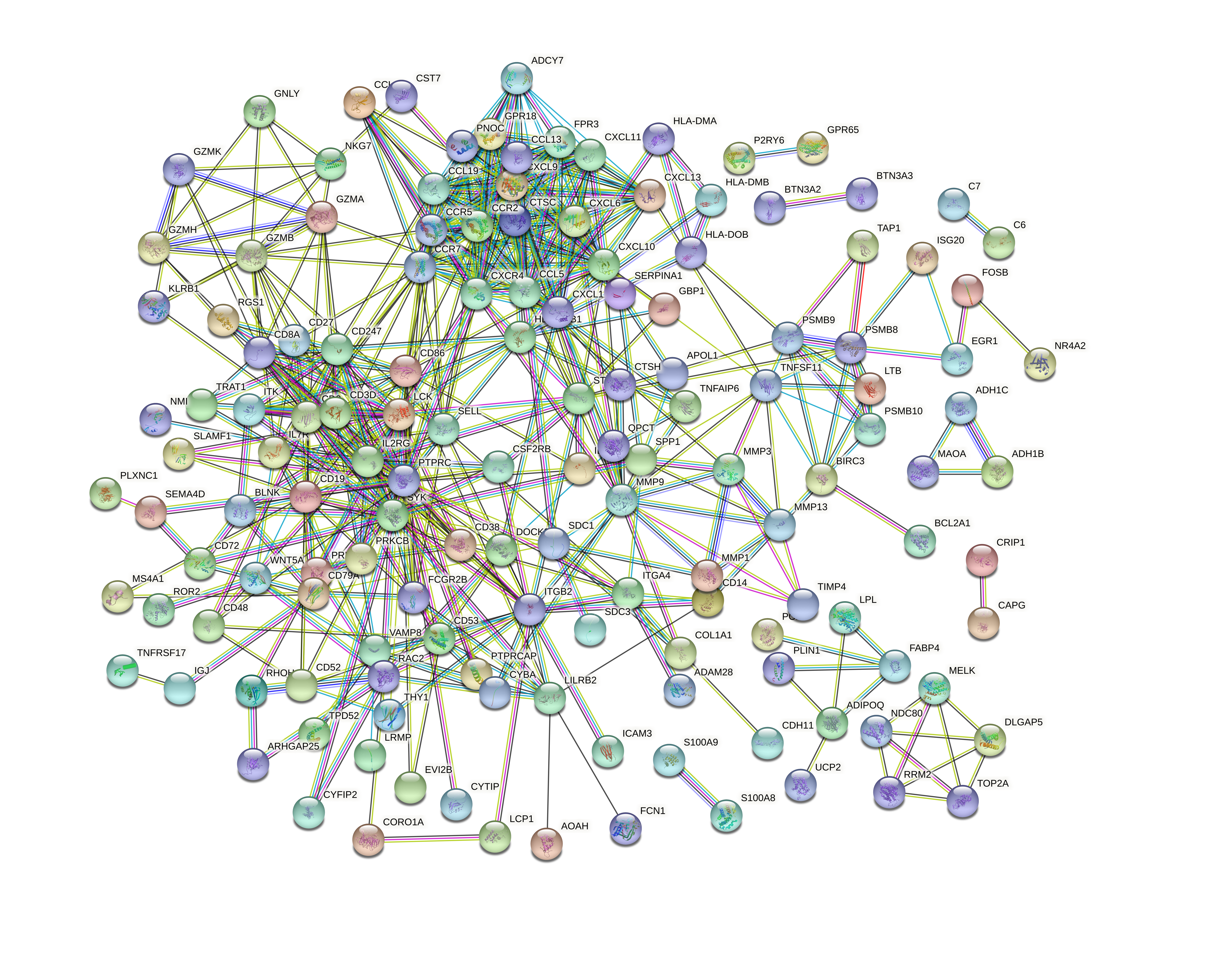

Supplement: Supplementary Figure 3 — PPI network results of DEGs by STRING. [file Image_3.png]
